# A Novel Experimental Approach for the Measurement of Vibration-Induced Changes in the Rheological Properties of Ex Vivo Ovine Brain Tissue

**DOI:** 10.3390/s24072022

**Published:** 2024-03-22

**Authors:** Rebecca L. Lilley, Natalia Kabaliuk, Antoine Reynaud, Pavithran Devananthan, Nicole Smith, Paul D. Docherty

**Affiliations:** 1 Department of Mechanical Engineering, University of Canterbury, Christchurch 8140, New Zealand; rebeccalilley3@gmail.com (R.L.L.); natalia.kabaliuk@canterbury.ac.nz (N.K.); antoine.reynaud@treefrog.fr (A.R.); pav.devananthan@pg.canterbury.ac.nz (P.D.); 2 Biomolecular Interaction Centre, Christchurch 8140, New Zealand; 3 École Nationale Supérieure de Mécanique et des Microtechniques, 25000 Besançon, France; 4 Department of Electrical Engineering, University of Canterbury, Christchurch 8140, New Zealand; smith.ni@icloud.com; 5 Institute for Technical Medicine, Furtwangen University, 78120 Villingen Schwenningen, Germany

**Keywords:** traumatic brain injury, brain tissue, rheology, mechanical fatigue

## Abstract

Increased incidence of traumatic brain injury (TBI) imposes a growing need to understand the pathology of brain trauma. A correlation between the incidence of multiple brain traumas and rates of behavioural and cognitive deficiencies has been identified amongst people that experienced multiple TBI events. Mechanically, repetitive TBIs may affect brain tissue in a similar way to cyclic loading. Hence, the potential susceptibility of brain tissue to mechanical fatigue is of interest. Although temporal changes in ovine brain tissue viscoelasticity and biological fatigue of other tissues such as tendons and arteries have been investigated, no methodology currently exists to cyclically load ex vivo brain tissue. A novel rheology-based approach found a consistent, initial stiffening response of the brain tissue before a notable softening when subjected to a subsequential cyclic rotational shear. History dependence of the mechanical properties of brain tissue indicates susceptibility to mechanical fatigue. Results from this investigation increase understanding of the fatigue properties of brain tissue and could be used to strengthen therapy and prevention of TBI, or computational models of repetitive head injuries.

## 1. Introduction

Traumatic brain injuries (TBIs) are prolific amongst high-impact-sports players, with some collegiate football players amassing over 1000 head injuries in a season [1]. Reports of depressive, anxious, and aggressive behaviours and cognitive delays have emerged from people with increased subjection to repeated TBIs, including high impact sports players, military personnel, and sufferers of physical abuse [2,3,4,5]. Despite regular subjection to TBIs, understanding of the neurological effects of repeated head trauma and importance of return to play legislation in sports is lacking [6]. Undertaking experimental investigation of brain tissue mechanics may mature the understanding of the pathological effects of multiple impacts, and lead to improvements in TBI treatment and prevention.

Mechanical fatigue is a means of material failure under repetitive application of stresses and strains [7]. Repetitive TBI (rTBI) and the cumulative effects of multiple sub-concussive head impacts can potentially be mimicked by subjecting the brain tissue to cyclic loading. Measuring the response of brain tissue to such loading may inform the understanding of the injury mechanism underlying rTBIs.

rTBIs have been shown to lead to cumulative detrimental health effects [8,9,10,11,12]. The effects of rTBIs on long term health and performance of individuals, particularly athletes, who can sustain numerous impacts to the head throughout a season, are the subject of ongoing TBI research, mainly due to potential long-term health detriments [8,12,13,14,15,16]. The impacts some athletes sustain can number in the hundreds to over 1000 a season [17]. The examples of afflictions that appear to have a link to rTBIs are Alzheimer’s disease, dementia, and depression [8,11,16,18,19,20,21,22].

A secondary head injury before the clearing of the symptoms of the first was linked to more severe health consequences compared to the primary injury, known as the Second Impact Syndrome (SIS) [23,24,25,26,27]. The studies claimed this might have been caused by an increase in extracellular potassium concentration and subsequent hypermetabolism that causes the brain to be more vulnerable to a second head injury. The controversy of SIS was reported by [27,28,29], based on the lack of consistent evidence among the collective case studies that were reviewed.

The biomechanical effects of repeated concussive and sub-concussive impacts on individuals were examined in several studies [9,30,31,32,33,34,35,36]. The effects of time between multiple impacts, impact location, magnitude, and direction of impacts are all considered factors that influence the medical health outcomes of such injuries [26,37].

Due to a lack of in-depth understanding of the biomechanical cranial impact and brain tissue’s biological response to mechanical TBI stimuli, it is challenging to establish deterministic relationships between impact mechanics and the injury threshold and severity. The link between the characteristics of the mechanical impact forces, the stresses and strains developed in the brain as a result of its movement within the cranial cavity at impact, and their corresponding mechanobiological responses (i.e., inflammation, oedema, amyloid plaque formation, and neurofibrillary tangles (NFT)) is of interest [19,21,29,36,38]. These mechanobiological factors associated with the tissue response to injury have not been thoroughly studied. Additionally, the mechanobiology of brain tissue when subjected to repeated impacts while suffering from a prior injury is still not fully understood [11,13,18,19,26,29,31,33,38]. The knowledge gap extends further to mechanobiological responses to mild rTBIs (mrTBIs) and repetitive sub-concussions, as these occur over extended periods such as over a game season.

This experimental investigation sought to determine potential history dependence of the viscoelastic properties of ex vivo brain tissue. Though susceptibility to failure by fatigue has been determined experimentally for other biological tissues, including human tendons and porcine coronary arteries [39,40], cyclic testing of brain tissue has yet to be investigated experimentally. Clamping methods required for uniaxial stress testing are prone to sample destruction, particularly for soft biological tissue with small sample sizes. Rheological methods provide a means of measuring the viscoelastic properties of brain tissue under cyclic loading without sample manipulation complications. Rheology is the study of how materials flow and deform under the influence of external forces, encompassing both liquid and soft solid states. It explores the interaction between viscosity and elasticity, revealing the material’s response to stresses, strains, and deformation rates. It is particularly effective at measuring the response of viscoelastic materials such as soft tissues [41]. Rheology can also be undertaken in vivo via magnetic resonance imaging (MRI) [42,43,44,45]. MRI has been used to assess the brain’s kinematic response to very mild impacts in vivo [46]. However, MRI is not a suitable tool for assessing repetitive impacts.

Computational models exist for cyclic loading of soft collagenous tissue [47]. These were recently extended in an attempt to conduct Finite Element Analysis (FEA) of repeated brain trauma by Gerber et al. [31]. The computational constitutive model of the mechanical response of brain tissue to repetitive loading was determined parametrically due to a lack of experimental data for brain tissue. Measuring the history dependent viscoelastic properties of brain tissue could strengthen current computational analysis and understanding of the biomechanics of repetitive TBIs and sub-concussions. This study assesses the efficacy of a rheometer-based approach to measuring vibration-induced changes in the mechanical behaviour of ex vivo ovine brain tissue.

## 2. Background

### 2.1. Small and Large Animal Models of TBI

Sub-concussive events are those which inflict axonal or neuronal damage, yet show no clinical symptoms of concussion [48]. Consequently, these impacts are more likely to have cumulative effects in sports players and military personnel, as symptoms may not be immediately recognised. A study of 224 boxers found a correlation between the length of career and reported behavioural and cognitive deficiencies, including aggression and memory loss [49]. In situ, Bajwa [50] found that social passivity and depressive behaviours were observed amongst mice 90 days after receiving a mild concussive brain injury. This suggests that mild concussive injuries could lead to chronic mental health problems. Hoogenboom et al. found structural changes in rat brain structure after mTBI but did not observe significant behavioural changes when compared to controls [51]. Mice that received bilateral mild TBI exhibited levels of white matter disruption which exceeded those that received single mild TBI [52]. A significant cumulative effect of repeated mild concussive injuries in comparison with a single event was reported after inflicting mild brain injuries by dropping weights onto the skulls of mice [53]. These findings could further verify the hypothesis that repeated or cyclic loading on the brain increases susceptibility to more significant damage than a single loading case.

There is some scrutiny of the bio-fidelity of small animal models of TBI in humans. Comparing the neuropathology and cognitive capacity of rodents with humans has limitations. Vink discussed the importance of investigating large animal models of traumatic brain injury to further the translation from animal models to understanding human TBI [54]. An area of particular concern was modelling the gyrencephalic brain of humans on lissencephalic rodent brains, largely because the presence of gyri influences the degree of deformation and maximum stresses imposed upon mechanical loading.

Non-accidental head injury (NAHI), as seen with shaken-baby syndrome, was investigated by subjecting 7–10 day old lambs to 10 sets of 20 s periods of manual shaking [55]. It was found that axonal and neuronal damage was widespread in hemispheric white matter. In vivo studies allow for healing, behavioural and cognitive changes, inflammation, metabolism, and other factors to be investigated. Cernak [56] highlighted the importance of controllable and repeatable means of inflicting trauma in experimentations, investigating modern animal models of brain trauma which sought to understand holistic effects of a multifaceted injury method. They noted that a limitation of ex vivo studies is that, typically, only one means of primary trauma is inflicted. This provides a delineated understanding of the response to certain trauma mechanisms, though has less direct comparison to accidentally induced trauma.

### 2.2. Biological Fatigue

Gilpin axially loaded ring sections of porcine coronary arteries to rupture [40]. This enabled measurement of the ultimate tensile strength (UTS) of the elastin and collagenous tissue. The study found that 3 cycles were required to rupture the tissue at 80% UTS and 65,280 cycles were required at 28% UTS. Load cycles plots were interpolated from these data to enable predictions of failure for arteries under certain cyclic loading patterns. Gilpin also determined failure via histological analysis. Fatigued samples had visible regions of damage where rings of arterial fibres had separated prior to global failure.

Investigation of history-dependent properties of in vitro human tendon tissue in uniaxial stress reported decreasing values of loss and storage modulus after partial fatigue at 10 and 20% of the measured UTS of 100 MPa [39]. Similarly, ex vivo fatigue testing of wallaby tendons in uniaxial loading reported decreasing elastic properties with successive loading before rupture [57]. Gilpin noted similar behaviour of increasing strain for a given stress upon subjection to cyclic loading of porcine arterial samples, characteristic of materials with fatigue sensitivity. This indicates that fatigue in biological tissues can be identified as the degradation of elastic solid mechanical properties upon application of successive loading cycles.

Gerber used FEA to model the potential cumulative damage from multiple TBIs [31]. The brain was modelled as a fibre-reinforced composite material that degraded with cyclic loading. The elastic degradation and eventual failure were modelled after previous studies in vitro on tendons, intervertebral discs, and bio-prosthetic heart valves. Stress softening has also been identified to occur upon initial loading of brain tissue. Gerber identified that a key transition in tissue fidelity occurs when the linear elastic limit of a sample is exceeded and loss modulus becomes non-constant. They claim that this is indicative of the ‘progressive rupture of the filamentary structure’. Computational cyclic loading of collagenous bio-prosthetic heart valves described the mechanism of collagenous material failure as having three distinct phases: fibril extension, uncrimping which increases tissue stiffness, and finally the damage region as interfibrillar bonds break [47]. Fatigue was identified after a negative stress, and permanent loss of elasticity in the plastic region and therefore deformation was observed at zero loading.

There are currently no published experimental studies that determine the changes in mechanical properties of brain tissue due to cyclic or repeated loads. New measurement techniques are required to provide direct information to computational studies and reduce the level of inference of brain tissue mechanical fatigue currently required. Information on the fatigue characteristics of brain tissue would ultimately provide important validation for computational brain fatigue simulations that seek to determine the effect of multiple TBI and sub-concussive incidents.

## 3. Methods

**Figure 1:**
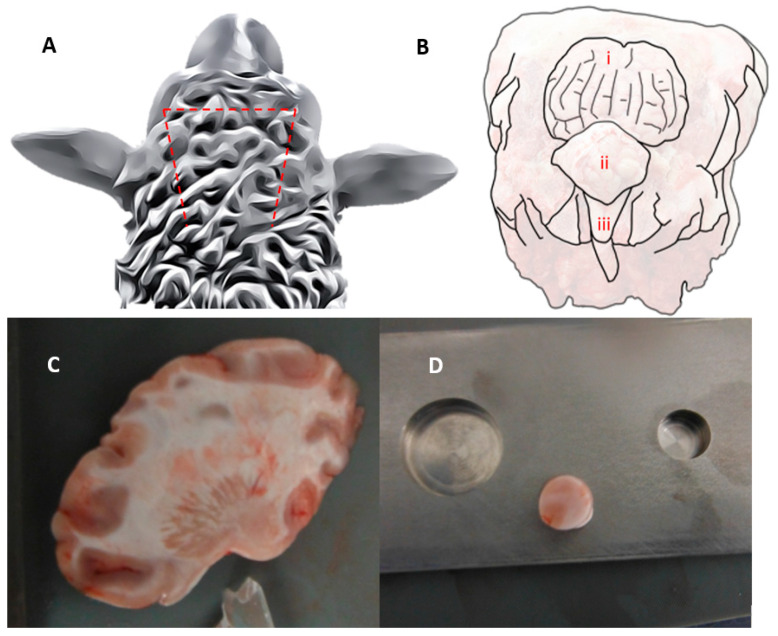
(**A**) Three sawing pathways to expose sheep brain from skull. (**B**) Sheep head with exposed parietal lobe (i), cerebellum (ii), and spinal cord (iii). (**C**) Sagittal slice of right cerebral hemisphere. (**D**) Ø10 mm cylindrical sample on stainless steel mould.

Abattoir ovine brain tissue was used in this study. To ensure consistency during this validation study, all brain tissue was obtained from animals that were assumed to be healthy and free from any known incidence of TBI. The brain tissue was extracted from a sheep skull with the help of a bone saw, making a coronal cut anterior to the ears, and two bilateral cuts toward the occipital condyle (Figure 1A). This resulted in the removal of an approximately triangular section of skull, revealing the cerebellum, spinal cord, and parietal lobe (Figure 1B). Two 12 mm sagittal slices (Figure 1C) were made in the left and right hemispheres using a fileting knife and two 6 mm stainless-steel cutting blocks. A Ø10 mm cylindrical sample was taken from the frontal cortex of each hemisphere using a die punch, avoiding the corpus callosum. The punch was rotated slowly to reduce compressive force. Cylindrical samples were put bottom-end first into a 5 mm deep, 10 mm in diameter hole that was milled from a stainless-steel cuboid (Figure 1D). A microtome blade was used to cut across the top of the template. Samples were then submerged in phosphate-buffered saline.

An Anton Paar MCR 302 Rheometer (Anton Paar, Graz, Austria) was used to undertake rheological testing. This rheometer is equipped with a 360° capacitive sensor, ensuring precise normal force measurement at a resolution of 0.005 N through a non-contacting and fully integrated bearing system. The displacement transducer design relies on a high-resolution optical encoder, guaranteeing accuracy in capturing minute changes (0.5 μrad) at angular frequencies as low as 10^−7^ rad/s. Additionally, the instrument incorporates the TruGap™ feature to reliably detect the real measuring gap, while temperature control is achieved through an Electrically Heated Temperature Device (0.01 °C accuracy). Furthermore, the device’s Combined Motor Transducer (CMT) is capable of imparting as little as 0.500 nNm of torque to the sample in oscillatory testing [58].

**Figure 2:**
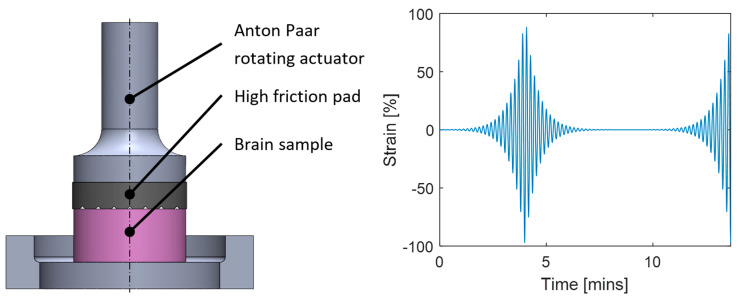
Sample loading diagram (**left**) and strain amplitude sweep diagram (**right**). Note that only 1.5 of 20 sweeps are shown, and the frequency has been lowered significantly to allow visualization.

After initializing the rheometer and custom 10 mm parallel testing plate, samples were loaded onto the Peltier plate, maintained at 37 °C. Rotational shear strain amplitude sweep tests were undertaken at a frequency of 3 Hz with ~7000 strain cycles per sweep evenly distributed on a logarithmic scale from 0.1% to 100% strain (Figure 2). Successive amplitude sweeps had a 100 s preloading phase which was applied prior to the first loading cycle to achieve 50 mN normal force, to be maintained for the remainder of the testing. Twenty amplitude sweeps were undertaken with a 100 s recovery period between successive cycles, as noted as best practice by Hrapko [59]. Samples were moistened by applying approximately 1 mL of phosphate buffered saline solution around their perimeter in the recovery period of each test, avoiding letting the liquid form a meniscus at the upper rheometer plate.

The rheometer recorded shear stress, rotational shear strain, storage modulus, complex shear modulus, normal force, and the gap between the parallel plates six times every decade in shear strain percentage. Recorded elastic and loss modulus and stress data were then plotted against shear strain to identify trends with successive cycles. The changes in viscoelastic properties of brain tissue after repeated cycles were of interest. Feng published a review of multiple rheological studies of brain tissue [60]. Among the studies reviewed by Feng, Bilston reported storage and loss moduli of 800–2000 Pa and 300–500 Pa, respectively, for bovine unspecified white matter at 1, 5, and 20 Hz [61]. They also found storage moduli of 1000–1500 Pa and loss moduli of 600–900 Pa for bovine corpus callosum and corona radiata samples at 0.01–20 Hz [62].

## 4. Results

**Figure 3:**
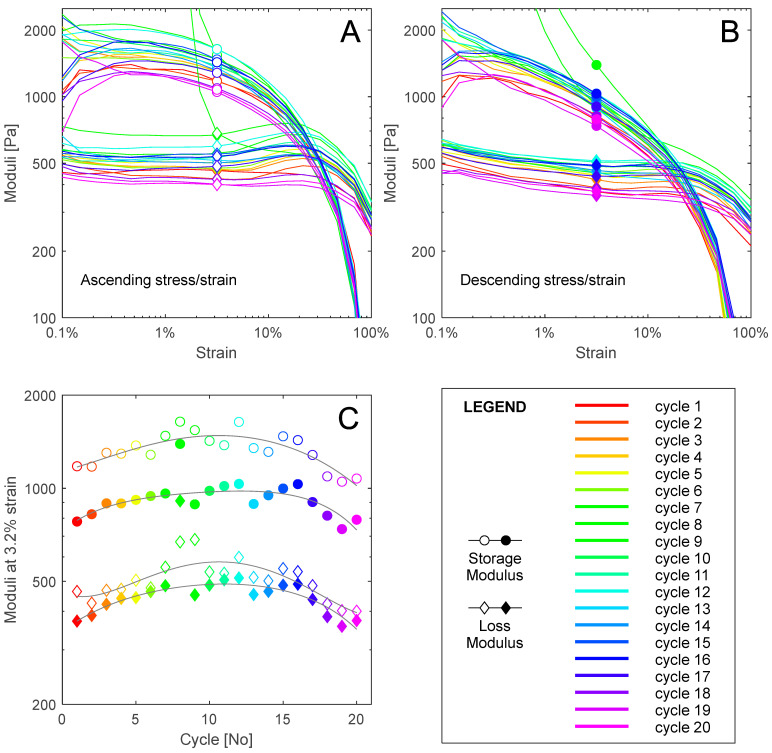
The evolution of storage and loss moduli against shear strain of the neurological tissue sample across 20 strain amplitude sweep cycles at 3 Hz. Subplot (**A**) shows the changes in moduli during the ascending phase of the amplitude sweep. Subplot (**B**) shows the changes in moduli during the descending phase of the amplitude sweep. Subplot (**C**) shows the evolution of moduli at 3.2% strain across the subsequent amplitude sweeps.

An excess of 20 samples were tested from 10 ovine brains in determining the best sample preparation and rheological testing protocol for cycle loading. For samples tested at 3 Hz, at 1% strain of the first three cycles, storage modulus values of 800–2000 Pa and loss modulus of 400–600 Pa were recorded consistently. This fell within agreeable ranges from a meta-analysis by Feng [60]. A sample taken from the frontal cortex of the right hemisphere with an approximate white matter composition of 65% was subjected to 20 rheological amplitude sweep cycles at 3 Hz, resulting in a total of approximately 28,000 cycles at varying strains. The viscoelastic properties of the sample with successive loading cycles are presented in Figure 3. The following analysis excludes data from cycle 9 where uncharacteristically high storage and loss moduli values occurred between 0.1% and 3% strain, as the sample was not appropriately moisturised.

**Figure 4:**
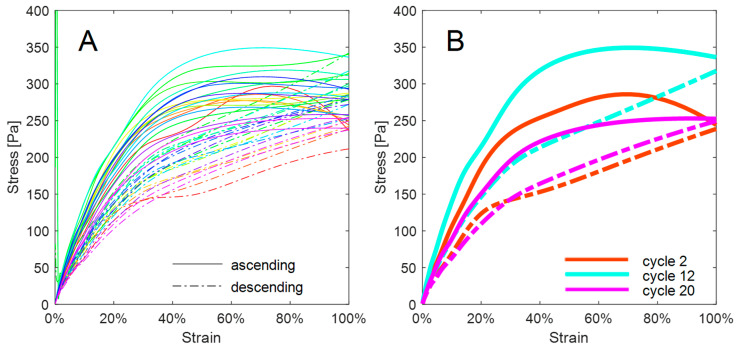
Shear stress against strain of the neurological tissue sample across 20 amplitude sweep cycles at 3 Hz. Note the hysteresis on increasing and decreasing amplitude sweeps. Subplot (**A**) shows all data, while subplot (**B**) shows data from the 2nd, 12th, and 20th amplitude sweeps alone to enable a better view of the changes over time. Figure 4 uses the same colour legend as Figure 3.

Elastic properties decreased with increasing strain, while the viscous property of loss modulus was approximately constant for a given cycle. Values for storage modulus decreased with successive cycles from a maximum value at 1% strain of 2008 Pa to a minimum of 957 Pa. A stiffening from 1521.8 Pa at 1% strain in ascending cycle 3 to the maximum at cycle 8 was observed before the degradation of elastic properties observed in comparing data from cycles 8 through to 20. The elastic and viscous response of the tissue was lower in descending amplitude sweeps, possibly because of the lack of relaxation time between ascending and descending sweeps. Ascending storage modulus with subsequent cycles at 3.2% strain shows a clear stiffening response before softening beyond cycle 8. The shear stress versus strain hysteresis response through increasing and decreasing amplitude sweeps for each cycle is presented as Figure 4.

The hysteresis loops for each cycle show a lower response in stress for a given strain percentage in the downwards amplitude sweep. The highest stress of 349.11 Pa was measured in cycle 12 at 68.2% strain, while the lowest stress in an upwards sweep of 202.8 Pa at the same strain was measured in cycle 20. Isolated hysteresis loops of cycles 2, 12, and 20 show the stiffening response of the sample before degradation of elastic properties. The hysteresis loops also became tighter beyond cycle 12.

## 5. Discussion

This study presents a novel approach to measuring changes in viscoelastic properties in neurological tissue due to repeated amplitude sweeps. The method employs a typical rheological apparatus, with specific sample mounting and loading protocols. A decreasing storage modulus and stress response against shear strain were identified in Figure 3 and Figure 4 after an initial stiffening response. Successive loading cycles induced degradation of the elastic properties of the brain tissue, indicating that the methodology successfully captured history dependent ex vivo brain tissue behaviour. Decreasing stress hysteresis magnitude with successive loading cycles followed fatigue behaviour similar to that observed in collagenous biological tissues [47]. Extension and progressive failure of fibres and interfibrillar bonds results in a loss of elasticity, and a similar response was seen in the decreased storage modulus of ovine brain tissue after exposure to cycle loading, indicating the degradation of tissue structural integrity (Figure 3).

Studies of tendons and porcine heart valves indicate that history dependence of biological materials can be identified as the degradation of elastic solid mechanical properties with successive loading cycles [39,40,57]. Such properties include storage modulus and measured stress for a given strain. A degradation of storage modulus (Figure 3 and Figure 4) was noted for ovine brain tissue beyond cycle 12. The stiffening response observed in Figure 3 and Figure 4 before softening could indicate similarity between the history-dependent properties of soft brain tissue and collagenous material. Gilpin noted a phase of stiffening response of collagenous materials to cyclic loading as fibrils uncrimp [40]. From their analysis, onset of fatigue could be determined experimentally in future experimentation if a negative stress is recorded with zero loading.

While fatigue characteristics have been investigated by Schechtman on ex vivo human tendon [39], Wang on ex vivo wallaby tendon [57], and Gilpin on ex vivo porcine arteries [40], no currently published studies have undertaken this fatigue analysis in ex vivo large mammal brain tissue. The results presented in Figure 3 and Figure 4 show similar behaviours to observations of tendon and artery cycle loading mechanics [39] and previously reported low-cycle viscoelastic properties of brain tissue [60]. In the absence of established gold-standard brain mechanical fatigue measurement techniques or previous research into mechanical fatigue properties of ex vivo brain tissue, comparison of these tendon and artery studies provides the best possible validation.

The rheological methods employed in this study allowed for cyclic loading of brain tissue to be undertaken. Typical methods used to measure history dependence of mechanical properties induce cyclic uniaxial stress in samples. However, it is difficult to determine how to clamp soft biological tissues, including brain tissues, without damaging the sample, or reducing the viable sample size such that it would be difficult to determine a representative region for testing. In particular, each ovine brain hemisphere only allowed five Ø10 × 5 mm samples due to the complexity of brain anatomy and requirement for relatively homogenous material. It does not seem possible to extract the typical dog-bone shape often used in tensile tests. The rotational shear implementation of the rheometer provided a controlled environment and a means of repeatable strain application. In particular, the resolution of the rheometer is orders of magnitude smaller than the variability in rheological properties observed across sweeps. Thus, it should be expected that measurement precision is immaterial to the variability observed in the rheological parameters.

The rheological methods utilised were time intensive, requiring approximately 4 min per amplitude sweep and ~4 h for the entire 20 cycle test. Hence, degradation of sample properties may be partially caused by time post mortem. This was minimised by replicating in vivo conditions; testing at 37 °C and moisturising the sample with a phosphate-buffered saline solution. A consistent limitation of ex vivo testing is the inability to perfectly replicate in vivo conditions and the repair mechanisms of living tissue. However, this investigation has increased understanding of brain tissue as a viscoelastic material upon exposure to cycle loading. Further investigations could be undertaken as a result of these findings to understand the response of in vivo brain tissue to cyclic loading through repetitive TBI and sub-concussive exposures.

The results from this investigation indicate the validity of exploring the history-dependent response of the brain as a complex viscoelastic and non-linear biological tissue. Further work can be undertaken to create a stress-versus-cycle (S-N curve) tissue response by first determining a mean UTS of the biological tissue and subjecting samples to cyclic loading at specified fractions of the UTS. This research verifies the predictions made from computational studies about the susceptibility of brain tissue to fatigue and allows for further experimental studies to be undertaken to strengthen existing computational models of failure of soft biological tissue and response to TBIs [31,47]. Histological methods should be employed to understand microstructural mechanisms of failure. Cell death, diffuse axonal damage, and neuronal damage in brain samples subjected to TBIs [48,63] could be investigated by staining with Cresyl violet, immunohistochemistry of β-amyloid precursor protein, and staining silver, respectively [64,65]. Furthermore, the rheological methods used in brain tissue fatigue investigation could potentially allow cyclic rheological measurement of other soft biological tissues that are unsuited to tensile testing.

## 6. Conclusions

This research has identified a lack of literature exploring the cyclic response of brain tissue. This paper described a novel method to enable rheological measurement of ex vivo brain tissue under cyclic loading. Changes similar to those observed in viscoelastic response of biological tissue, such as tendon and artery walls, under cyclic loading were observed in this study of brain tissue. The method was successfully validated against the limited existing low cycle mechanical characteristics of brain tissue. This methodology may facilitate more accurate models of brain tissue response to repetitive TBI and cumulative sub-concussions that may enable improved treatment or protections for those at risk of multiple head injuries.

## Data Availability

Data can be made available upon request to the corresponding author.
